# Clinical Evaluation of a Microwave Device for Treating Axillary Hyperhidrosis

**DOI:** 10.1111/j.1524-4725.2012.02375.x

**Published:** 2012-03-27

**Authors:** H Chih-Ho Hong, Mark Lupin, Kathryn F O'Shaughnessy

**Affiliations:** * Department of Dermatology and Skin Science, University of British ColumbiaVancouver, British Columbia, Canada; † Miramar Labs, IncSunnyvale, California

## Abstract

**Background:**

A third-generation microwave-based device has been developed to treat axillary hyperhidrosis by selectively heating the interface between the skin and underlying fat where the sweat glands reside.

**Materials and Methods:**

Thirty-one (31) adults with primary axillary hyperhidrosis were enrolled. All subjects had one to three procedure sessions over a 6-month period to treat both axillae fully. Efficacy was assessed using the Hyperhidrosis Disease Severity Scale (HDSS), gravimetric weight of sweat, and the Dermatologic Life Quality Index (DLQI), a dermatology-specific quality-of-life scale. Subject safety was assessed at each visit. Subjects were followed for 12 months after all procedure sessions were complete.

**Results:**

At the 12-month follow-up visit, 90.3% had HDSS scores of 1 or 2, 90.3% had at least a 50% reduction in axillary sweat from baseline, and 85.2% had a reduction of at least 5 points on the DLQI. All subjects experienced transient effects in the treatment area such as swelling, discomfort, and numbness. The most common adverse event (12 subjects) was the presence of altered sensation in the skin of the arm that resolved in all subjects.

**Conclusion:**

The device tested provided efficacious and durable treatment for axillary hyperhidrosis.

The prevalence of axillary hyperhidrosis in the United States has been estimated at 1.4%,^2004^ which amounted to more than 4.3 million people in 2011. Although there are several different treatment options,^2007^ only surgical modalities have been capable of conferring a permanent solution. A novel microwave device was developed^2012^ and has been cleared by the Food and Drug Administration as a noninvasive method to treat axillary hyperhidrosis. A prior randomized, blinded, multicenter study proved the efficacy of an earlier-generation device that implemented microwave technology.^2012^ The purpose of this present study was to evaluate a newer-generation device that delivers faster treatment times and to further assess efficacy and safety. This study was meant to provide information on device optimization, particularly with respect to energy levels, numbers of treatments, and timing of procedures.

Microwaves heat by physical rotation of dipole molecules. Because ionic water has a high dipole moment, and fat has a low dipole moment, there is relative selectivity to the water-rich dermis and sweat glands, with less absorption in the subcutaneous layer. Applying concurrent cooling to the upper dermis restricts the heat to a small zone near the interface, where the sweat glands are located. Although eccrine glands are the primary target in treatment of hyperhidrosis, this microwave device can also affect the odor-related apocrine glands. In that respect, this study evaluated reduction of odor in addition to sweat reduction.

## Materials and Methods

### Patients

Thirty-one adults with primary axillary hyperhidrosis were enrolled in a single-group unblinded study at two centers. All subjects had primary axillary hyperhidrosis evidenced by Hyperhidrosis Disease Severity Scale (HDSS) ratings of 3 or 4 and a gravimetric sweat assessment of at least 50 mg in 5 minutes in each axilla. Subjects were excluded if they had had surgery for axillary hyperhidrosis or botulinum toxin injections in the axillae in the last 12 months.

The study was conducted in accordance with the Declaration of Helsinki, and all subjects signed an Ethics Committee–approved informed consent before any study procedures.

### Sweat Assessments

**Figure 1:**
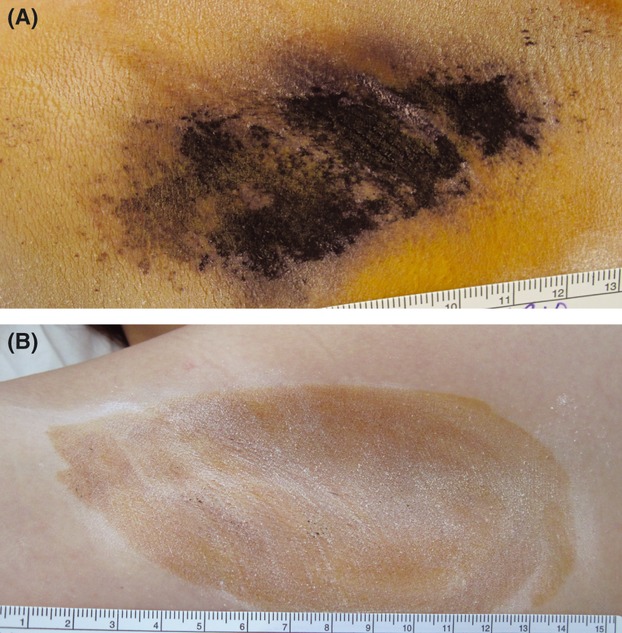
Starch-iodine photographs of the right axilla of subject at (A) baseline and (B) 6-month follow-up visit. Dark areas show active sweat glands. The subject had a Hyperhidrosis Disease Severity Scale (HDSS) score of 4 at baseline. At the 6-month visit, the HDSS score was 1, and the subject had a 91% reduction in sweat, as measured by the gravimetric assessment.

The primary method for assessing subjects' level of underarm sweat was subject-reported HDSS score.^2004^ Table 1 provides the definition of each of the four possible scores. A gravimetric (weight) assessment of sweat was used as a secondary measure. Subjects were required to be at rest in a normal-temperature room. Their axillae were wiped before the test, a preweighed filter paper (Whatman #541, 90 mm, Maidstone, UK) was placed in each axilla for 5 minutes, the filter papers were weighed again, and the difference in weight was calculated in milligrams. A secondary assessment used the 10-question, validated Dermatology Life Quality Index (DLQI).^2008^ Also, although not used for any quantitative study assessments, a starch-iodine test was employed in some treatment sessions to identify areas that still had active sweat glands and at follow-up visits for a visual assessment of sweat. An alcohol-based iodine mixture was wiped on the skin of the axilla, and then corn starch was sprinkled on the area and gently brushed to create a thin uniform coating. Any sweat that appeared turned black (Figure 1). Histologic samples from punch biopsies at baseline and after treatment were obtained if subjects agreed.

**Table 1:** Hyperhidrosis Disease Severity Scale (HDSS) Definition: How Would You Rate the Severity of Your Hyperhidrosis?

HDSS Score	Definition
1	My underarm sweating is never noticeable and never interferes with my daily activities
2	My underarm sweating is tolerable but sometimes interferes with my daily activities
3	My underarm sweating is barely tolerable and frequently interferes with my daily activities
4	My underarm sweating is intolerable and always interferes with my daily activities

To obtain overall subject assessment of the procedure, patients were asked to choose a satisfaction rating that described their evaluation of the procedure from the following categories: very satisfied, somewhat satisfied, neutral, somewhat dissatisfied, and very dissatisfied.

Finally, although the primary investigation was of the effect of the procedure on wetness (sweat) production, subjects were asked to provide their perception of underarm odor at baseline and at follow-up visits, given that the mechanism of action of the device might affect the apocrine and the eccrine sweat glands. Subjects were asked to provide their rating of their underarm odor as not noticeable, slightly noticeable, somewhat noticeable, noticeable, or very noticeable.

### Study Visit Schedule

Subjects attended a screening or baseline visit at which informed consent was obtained, and baseline sweat assessments were performed.

The treatment phase of the study started with the first procedure visit, at which the hair-bearing areas of both axillae were treated. As part of the procedure development, subsequent treatment phase visits were held approximately every 30 days for sweat assessments and safety evaluation; if the subject exhibited signs of sweat production (based on the answers to the subjective questionnaires or the starch-iodine test), another procedure session targeting the still-sweating areas was completed. At most, three procedure sessions were allowed, and all procedures had to be completed within a 6-month window.

Each procedure session included three steps: marking the axilla with a treatment template, injecting local anesthesia (1% lidocaine with 1:100 000 epinephrine) in a grid pattern throughout the indicated area, and applying the microwave treatment. The microwave-based device included integrated vacuum and cooling (miraDry System; Miramar Labs, Sunnyvale, CA) and allowed for a small range (±10%) of energy settings. Some variation in energy settings was explored to determine optimal parameters for the population being treated.

After all procedure sessions were complete, subjects attended follow-up visits at 30 days and 3, 6, and 12 months after their last procedure session.

### Study Efficacy Measures

Efficacy of sweat reduction was measured using all sweat assessments (HDSS, gravimetric assessment, and DLQI) and patient satisfaction. The primary overall efficacy measure was the percentage of subjects that reduced their HDSS scores from 3 or 4 at baseline to 1 or 2 at the follow-up visits. As a secondary measure, gravimetric scores for each visit were obtained by taking the average of the readings from the right and left axillae; the percentage reduction was calculated by dividing the change in gravimetric score (from baseline) by the baseline gravimetric score. Efficacy was measured by calculating the percentage of subjects who achieved at least a 50% reduction in their gravimetric score and by calculating the average percentage reduction for all patients. For the DLQI score, the average score for all patients at baseline was compared with the average score at the indicated follow-up visit. A further analysis calculated the percentage of patients that achieved at least a 5-point reduction in DLQI, which previously has been reported to be the change in the DLQI that represents a clinically meaningful improvement after therapy.^2007^ This last calculation was made only for patients who had a DLQI score of at least 5 at baseline. An overall patient satisfaction measure was calculated as the percentage of subjects who rated themselves as very satisfied or somewhat satisfied with the procedure results. An analysis for odor reduction was conducted by calculating the percentage of subjects that had not noticeable underarm odor at the follow-up visits.

### Statistical Analysis

Statistical analysis was conducted using SAS (SAS version 9.1; SAS Institute, Inc., Cary, NC). Exact binomial 95% confidence intervals were calculated for percentage values. For analysis of changes in DLQI for which paired values were available, a paired t-test was used to establish whether the average difference was more than 5 points. The McNemar exact test was used to determine statistical significance for the proportion of subjects reporting not noticeable underarm odor at follow-up visits. For any missing data points (e.g., for missed visits), the last observation carried forward method was used to fill in the missing values unless otherwise noted.

### Safety Assessments

At each study visit, subjects were asked a general question about their health. Reported procedure effects were categorized as Grade 0 if they were minor expected sequelae from the procedure (such as local swelling or bruising). Other events were categorized as Grade 1 (minor) to Grade 3 (severe). The duration of all events was tracked, and the investigators assigned the degree that the event was related to the procedure or device (none, remote, possible, probable, unknown).

## Results

Demographic information and baseline sweat assessment values for the group of enrolled subjects are shown in Table. Twenty-six of the 31 enrolled subjects completed study visits through 12 months of follow-up. Twelve subjects had two procedure sessions, and 15 had three procedure sessions within the allowed 6-month window. Four subjects had only one procedure session. (One subject achieved full sweat reduction with the one session, two declined further treatment because of side effects, and one declined because of lack of efficacy.)

**Table:** Demographic Characteristics and Baseline Sweat Assessments for the 31 Subjects

Characteristic	Value
Age, median (range)	33 (18–65)
Sex, *n* (%)	
Male	8 (26)
Female	23 (74)
Race, *n* (%)	
Caucasian	27 (87)
Asian	4 (13)
Body mass index, average, kg/m^2^	24.8
Baseline Hyperhidrosis Disease Severity Scale score, *n* (%)	
3	20 (65)
4	11 (35)
Baseline average gravimetric reading, mg/5 minutes	190
Baseline Dermatologic Life Quality Index, average	11.8

### Efficacy

The primary and secondary efficacy results for each follow-up visit are shown in Table 3, along with 95% confidence intervals. The primary efficacy HDSS result was 90% or higher as measured at all four follow-up visits. (See Figure for the distribution of HDSS values at each time point.) Additional analyses of HDSS at the final 12-month visit shows that 94% (29/31) of the subjects had at least a 1-point drop in HDSS and 55% (17/31) had a 2-point drop or greater in HDSS.

**Figure 2:**
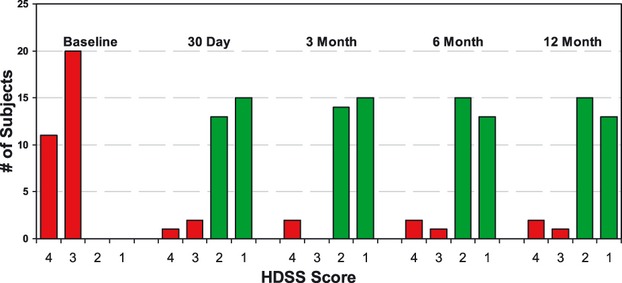
Distribution of Hyperhidrosis Disease Severity Scale (HDSS) scores at the different study visits. Ninety percent or more of subjects had a reduction to scores of 1 or 2 after treatment at all of the follow-up visits.

**Table:** Sweat Efficacy Assessment Results at Study Follow-Up Visits

Efficacy Measure	30 Days	3 Months	6 Months	12 Months
HDSS reduction to score of 1 or 2, *n* (%) [95% CI]	28 (90.3) [74.3–98.0]	29 (93.6) [78.6–99.2]	28 (90.3) [74.3–98.0]	28 (90.3) [74.3–98.0]
≥50% reduction in sweat (gravimetric), *n* (%) [95% CI]	28 (90.3) [74.3–98.0]	29 (93.6) [78.6–99.2]	28 (90.3) [74.3–98.0]	28 (90.3) [74.3–98.0]
Average reduction in sweat (gravimetric), % [*n* = 31]	83.1	82.3	82.1	81.7
Average DLQI score [*n* = 31]	2.5	2.7	3.1	3.0
Reduction in DLQI score, average [95% CI]*	10.4 [8.3–12.4]	10.2 [7.9–12.4]	9.6 [7.3–12.0]	9.9 [7.5–12.2]
Reduction of DLQI by ≥5 points, *n* (%) [95% CI]*	26 (96.3) [81.0–99.9]	24 (88.9) [70.8–97.7]	24 (88.9) [70.8–97.7]	23 (85.2) [66.3–95.8]

* Included only patients with a baseline DLQI of ≥5 [*n* = 27].

CI confidence interval; DLQI, Dermatologic Life Quality Index.

95% confidence intervals are shown in square brackets.

The efficacy measured as the percentage of subjects with a 50% or greater reduction in gravimetric assessment (90%, 94%, 90%, and 90% at the 30-day, 3-month, 6-month, and 12-month visits, respectively) further supports this result. The average reduction in sweat for all patients was 83%, 82%, 82%, and 82% at the 30-day, 3-month, 6-month and 12-month visits, respectively. Figure shows the per-patient reduction in sweat from baseline at the 12-month follow-up visit. The percentage of subjects who showed a reduction in DLQI score of at least 5 points was 96%, 89%, 89%, and 85% at the 30-day, 3-month, 6-month, and 12-month visits, respectively, and the average DLQI reduction was statistically significantly greater than 5 points (the average reduction was 10.4, 10.2, 9.6, and 9.9 points for the 30-day, 3-month, 6-month, and 12-month visits, respectively, *p* < .001 for all four values). The elimination of sweat glands can be seen in histologic samples that one subject provided. FigureA shows a sample taken at baseline, with the sweat glands clearly evident below the skin. FigureB shows a sample taken in the same axilla 105 days after the last treatment, with no sweat glands evident.

**Figure 3:**
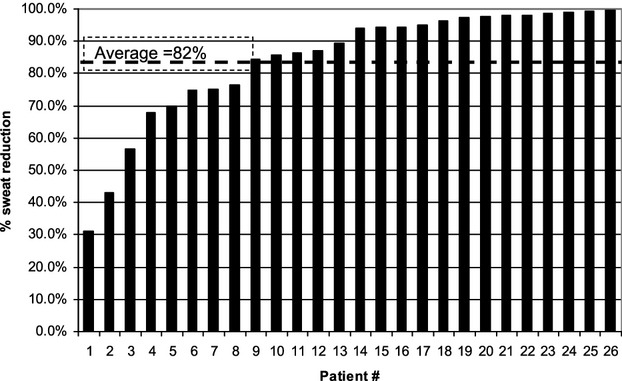
Individual patient percentage reduction in sweat as measured by gravimetric assessment comparing results at 12 months with baseline. Only the subjects who attended the 12-month visit (*n* = 26) are shown.

**Figure 4:**
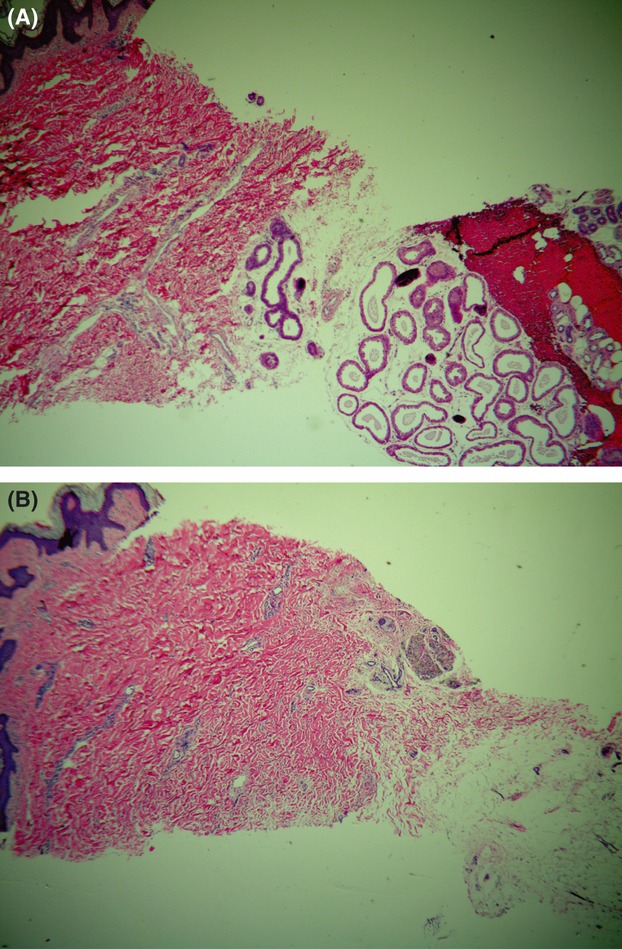
Histology samples show (A) baseline appearance of sweat glands present just under the skin and (B) sample taken from a different location in the same axilla after treatment. The post-treatment sample shows that sweat glands are no longer present under the skin. (Hematoxylin-eosin sample, magnification ×40.)

Ninety percent of subjects who completed the questionnaire (27/30) reported being very satisfied or somewhat satisfied at the 30-day follow-up visit. This percentage was 96% (27/28) at the 3-month visit, 93% (25/27) at the 6-month visit, and 88.5% (23/26) at the 12-month visit.

The data on subject-reported odor evaluation also showed that a large percentage of subjects who started the study with a self-reported underarm odor as noticeable (to any degree) found that their underarm odor was not noticeable after the treatment. The percentage of subjects who had not noticeable odor at baseline was 12.9% (4/31); at the follow-up visits the proportion was 67.7% (21/31), 71.0% (22/31), 74.2% (23/31), and 61.3% (19/31) at the 30-day, 3-month, 6-month, and 12-month visits, respectively (*p* < .001 at all time points).

### Safety

The reported Grade 0 events showed that a large number of subjects experienced mild procedure effects that typically lasted a few days to a week; the most common were edema (90% of subjects), redness and vacuum acquisition marks (87% of subjects), and discomfort (84% of subjects). The longer-term effects (all resolved) were altered sensation in the skin of the axillae (65% of subjects, median duration 37 days, range 4 days to 4 months) and palpable bumps under the skin of the axillae (71% of subjects, median duration was 41 days, two subjects had the effect at study exit). The duration for axillary hair loss (self-reported by 26% of subjects) was not calculated because, in the majority of affected subjects, it was ongoing at the time of study exit.

Procedure effects that were rated to have even a remote chance of being procedure related that were evident outside the axillae were seen in 18 (58%) subjects; 88% of the events were rated as mild. The most common effect seen (16 events in 12 subjects) was altered sensation in the skin of the treatment limb (median duration 50 days, range 6 days to 12 months), and all resolved. The second-most-common effect was swelling outside the axilla, in the arm or chest, (median duration 7 days, range 2–23 days). One subject experienced transient neuropathy of the left arm with associated muscle weakness after the procedure; the prognosis from the consulting neurologist was complete resolution. The subject showed improvement 6 months after treatment, after which she was lost to follow-up.

## Discussion

Primary axillary hyperhidrosis is a common problem with a significant effect on quality of life. Previous treatment modalities include topical antiperspirants, botulinum toxin injections, surgical interventions, and oral anticholinergic medications.

There has recently been the development of a novel microwave energy device that destroys eccrine glands at the interface of the deep dermis and subcutis, minimizing damage to surrounding tissue. An earlier-generation device was reported to be efficacious and safe in a randomized, blinded, multicenter study.^2012^ A newer-generation device was tested for safety and efficacy. Optimization of treatment parameters and treatment protocol was also assessed during the study.

After the failure of topical antiperspirants, injection with botulinum toxin is the most common intervention. In one study,^2005^ the HDSS efficacy was 85% (121/142) 4 weeks after treatment and 90% (115/128) 12 weeks after treatment (where efficacy was calculated as the percentage of subjects reaching an HDSS score of 1 or 2). Longer duration of effect was not measured in the study. A study with longer follow-up^2007^ reported efficacy of 75% 4 weeks after treatment (where efficacy was calculated as the percentage of subjects with a ≥2-point drop in HDSS) and an estimated Kaplan–Meier efficacy of 22% at 52 weeks. A similar analysis for the study in this report shows efficacy of 18 out of 31 (58%) at 30 days and essentially the same value (17/31, 55%) 12 months after treatment. These data illustrate the primary difference between botulinum toxin and microwave treatment, in that the effect of the botulinum toxin injections is temporary, with an average duration of 6.7 months,^2007^ and the microwave treatment has shown stable results through the last follow-up visit at 12 months.

At the visits 30 days and 3, 6, and 12 months after the allowed treatment series, 90.3%, 93.6%, 90.3%, and 90.3% of subjects, respectively had achieved the primary end point of an HDSS score of 1 or 2. Gravimetric analysis, a secondary end point, showed a reduction of sweat of 83.1%, 82.3%, 82.1%, and 81.7% from baseline at the same time points. The average reduction in the DLQI at the above-mentioned time points was 10.4, 10.2, 9.6, and 9.9, which is a dramatic change in DLQI. It is also significantly higher than improvements that represent a clinically meaningful change in DLQI after therapy for primary axillary hyperhidrosis.^2007^ Overall, patient satisfaction was high, with 90% of patients reporting satisfaction at each of the four follow-up time points through 1 year. The treatment also seems to affect subject-reported underarm odor, with a statistically significant increase in the percentage of patients who reported not noticeable underarm odor. The efficacy of the treatment did not seem to vary with the number of procedures.

Short-term adverse events related to the therapy were generally minor. Post-treatment edema, erythema, and discomfort in the treatment area were common and resolved quickly after therapy. Some patients experienced longer-lasting transient effects, such as altered sensation in or around the treatment area, papule and nodule formation in the axilla, and hair loss. Some subjects were still experiencing axillary hair loss when they exited the study. One patient experienced treatment-related neuropathy that was resolving at 6 months, after which she was lost to follow-up.

The miraDry system is a novel microwave energy device that can be used to treat axillary hyperhidrosis through selective heating of the lower layer of skin, where the eccrine and apocrine glands are located. Patient satisfaction with the procedure is high, and adverse events are typically transient and well tolerated. This system provides a durable, noninvasive alternative therapeutic modality for patients with this common and frustrating problem.
